# Momentary Working Memory and Momentary Activities in Healthy Older Adults: The Mediating Role of Affective States

**DOI:** 10.1093/geroni/igab046.2221

**Published:** 2021-12-17

**Authors:** Minxia Luo, Robert Moulder, Christina Röcke

**Affiliations:** University of Zurich, Zurich, Zurich, Switzerland

## Abstract

Research has shown cognitive ability in older age is associated with activity engagement, but little is known about what psychological mechanisms are linking the two constructs. This study investigates an emotional pathway, in which affective states mediate the temporal associations between momentary working memory and momentary activities in older age. We examined data from 153 healthy older adults aged 65 to 91 who completed a smartphone-based ambulatory assessment survey seven times a day over 15 days. In each assessment point, participants reported their momentary activities (e.g., social activities, mentally stimulating activities) and affective states (i.e., positive affect, negative affect) and took a working memory task. Initial results suggest that during an approximate time period of six hours (i.e., across three assessment points), working memory performance influences subsequent likelihood of social activity engagement. Moreover, positive affect mediates this temporal association. Results will be discussed in the context of cognitive aging research.

